# American Dental Association

**Published:** 1872-01

**Authors:** 


### ARTICLE IV.

### *American Dental Association.*

[CONCLUDED.]

The following Special Committee on Dental Education was appointed: Dr. S. B. Palmer, Syracuse, N. Y.; Prof. Homer Judd, St. Louis, Mo.; Dr. M. S. Dean, Chicago, Ill.

A further report by the newly-appointed committee was then read by Dr. Francis.

The report claimed to be only a few simple suggestions. It is essential that all who practice a profession so indispensable to the requirements of the age—when health, comfort, and even life depend upon the judgement and skill of its members—should be thoroughly fitted for their mission. With the abundant opportunities, it is time that the day of empiricism had passed. Educational institutions are conveniently located; text-books are accessible to all; periodicals come each month well freighted with thoughts and suggestions; organized dental societies in nearly every State and city afford opportunity for interchange of thought and comparison of investigation ; and in view of all this it would seem that there is little excuse for plodding in darkness, unaided by science. No profession has made such strides as ours; yet, as compared with other professions, we are far in the background. This should not be the case; and the fnture will testify that it will not be the case. Among the the twelve thousand or fourteen thousand dentists of this country, are hundreds, yes thousands, who have had little or no preliminary instruction. Probably not one-quarter of this number read the journals, and are familiar with text books. We know that in families or communities where newspapers and books are ignored we find ignorance and superstition. On the same principle, are not those of our profession who discard books and their teachings in the slough of ignorance.

The number of members of societies cannot be ascertained, but it is vaguely estimated that only about one-tenth of those calling themselves dentists are members of any dental organization; and of this small number scarcely one in three take a genuine interest in their societies, such as preparing essays and participating in discussions.

We have nine dental colleges in the United States in a healthy condition. We regret that their sphere cannot be more widely extended. We would make it a legal requirement for all new-comers to receive the benefit of their teachings. While charlatans and empirics drag at our skirts, so long must we expect to share in the odium that falls upon their heads. We hope the time is not distant when every State will require every practitioner to give evidence of his ability and fitness, as to principles and skill. A law of this character need not be unjust. If a board of censors should be required to pass upon every applicant, what a change would soon be effected. Men in active practice, if allowed three or five years to fit themselves to pass the required examination, would not only in the end be themselves benefited but their patients would be doubly benefited. Even in a mercenary view of the matter, the more valuable will be the dentist's services to his patient, and the greater the remuneration received for the higher skill employed.

Respecting students, the Committee suggest that while the value of private instruction is recognized, the importance of more extended scientific knowledge of underlying principles that is thus obtained cannot be overlooked, and they recommend that the profession generally impress upon their students the importance of pursuing a college course. In addition they would suggest that the colleges as well as private instructors, decline to receive students whose moral character is tainted with vice, or who does not possess a fair academic education. Then, properly equipped and fitted for their duties, the student may move forward in the path of usefulness and honor, receiving the reward for his toil.

Prof. McQuillen said that his conceptions of what a denist should be are of an exalted character. He should be a gentleman. If not one by nature, then those characteristics should be cultivated. He should be an educated man as his calling brings him constantly in contact with persons of education and refinement. He thought a collegiate education should be made compulsory upon all who propose to enter the profession, and all men would be able to get it if they had the will. The advantages of a compulsory education were exemplified in Europe during the late war, in the grand movements of Bismarck and Von Moltke. It is all nonsense for a man to say he cannot get a collegiate education, who has hands, head and health.

Dr. Atkinson. Medicine owes nearly every item of its truth to efforts of ignorant minds empirically put forth. I have been engaged in teaching twenty-five years, but have scarcely satisfied an ambition of my soul. I would go further in this matter than any who have yet spoken. I would have a man a graduate of schools before he can matriculate in dental knowledge. Fault-finding passes for criticism. Education is a serious cataloguing of mental concepts. "Dirt is only matter out of place." Let us speak forth our inmost convictions with truth and soberness on all occasions.

Dr. Walker indorses all that has been expressed by Drs. Atkinson and Francis. Charlatanism is not confined to those who are not graduates. We must have legal enactments to control this matter.

Dr. Crouse likes the idea of legislation, but if we pass such stringent laws, what are we going to do for office-boys? The ignoramuses are crowded into the profession in this way. The meanest men we have are graduates. Get the right kind of timber before you build your house. Competition among colleges leads to too slight discrimination as to the qualifications of graduates. Colleges may be benefitted by criticism. Men of the right kind of timber will work their way without means. There are too many colleges and journals—spreading things too much, and inducing too much competition. Adjourned.

Fourth Day—*Afternoon Session.*

Called to order by the President. Minutes read.

The Chair announced as the Publication Committee, Drs. Cushing and Forbes.

The report from the special committee on the Barnum testimonial was called for, and Dr. Cushing presented the medal, which he said was the report, and would speak for itself, and so far as its artistic merits were concerned, would bear close inspection. The medal was accepted, and placed in the hands of Dr. Cushing for delivery to Dr. Barnum.

Dr. Goddard moved to reconsider the vote by which the amendment offered by him last year relative to time of meeting was rejected. After discussion, the motion to reconsider was carried.

The original motion was verbally amended, the amendment carried, and the resolution as amended then put to vote and lost.

A resolution of thanks was passed to Drs. Allport, Cushing and McKellops, for procuring and presenting to this association the beautiful medal to be presented to Dr. Barnum, as a testimonial for the free gift of the rubber dam.

The report of the Publication Committee was then read by Dr. Dean.

The report stated that the committee had, in accordance with their instructions, employed the publishers of the *Dental Register* (they making the most favorable offer) to issue three hundred copies of the Transactions, in paper covers, which with the less number printed, and the mode of publicotian, effected a saving to the association. But the committee, without reflecting upon the publishers, did not recommend the future adoption of this plan, as the small saving was not commensurate with the disadvantages. The supervision of the work requires an attention quite impossible when it is done at a different place from the residence of the committee. The practice formerly adopted of transmitting the reports of each speaker to him for revision, resulted in its return so altered as to be scarcely recognized. This renders the Transactions more complete and scholarly, but tends to encourage a reckless style of debate. The committee have hit upon a compromise between this plan and that of publishing as reported. The reporter will, during the session, read over his notes to each speaker, giving an opportunity to correct errors while the subject is fresh : thus accuracy may be secured. The able stenographic reporter who was employed last year was secured for the present session. The charts accompying the report on Dental Pathology were engraved at the expense of the chairman of that committee. The accounts of the committee show an amount paid for printing Transactions, of $204 ; reporting, $80 ; sundries, $17 ; and a balance due to the committee of $4.07.

The Publication Committee were, on motion of Dr. Judd instructed to exercise their constitutional right of revision, in reference to the essays and discussions, excluding such matter as they deem unworthy of publication.

A motion to appoint a delegate to the Southern Dental Association was adopted.

The committee appointed for the purpose, reported resolutions of condolence in relation to the death of Drs. Peebles and Willis.

Report of Committee on Dental Literature was read by Dr. Cushing.

The report considered it the duty of the committee to endeavor to advance the interests of the profession by fair and judicious criticism upon what had been written ; and disclaimed any intention of personality in what might be said, all criticism being in the kindliest spirit, and should be acceptod in the same spirit.

The periodical literature of the profession claims the first attention. We are always in advance of text books, and must depend on the journals and transactions of societies for the most advanced knowledge ; without them we should soon be behind the age. To say that our journals are all that they should be, would be far from the truth ; but considering how poorly they are sustained, and at how much personal sacrifice they are conducted, we wonder that they are as valuable as they are. A marked improvement is perceptible, however, during the past two years. It is unfortunate that they cannot exclude from their pages much that appears in them; if we ask why they cannot, the only answer is—first, that those who can write well do not; and second, that the meagreness of the support by subscription hampers the conductors, so that they cannot be as independent as they ought. As a remedy, let every man who is competent, make it a part of his religion to furnish two or more papers annually, requesting the editor to revise or exclude, if needful; and, next, let every man who takes but one journal subscribe for all the rest—and if he takes none, subscribe for all at once. The editors cannot be held altogether blameless: they should more carefully revise what is sent them, suggest to authors such revisions as seem desirable, and thus improve not only the journals, but the future efforts of writers. The transactions of societies deserve mention. That of this body will compare favorably with those of former years. The Illinois State Dental Society has for three years published its Transactions, which we think will bear worthy mention. The committee commend this plan as one which, if pursued, would result in great benefit—not only by preserving valuable matter, but by improving both writers and speakers, and in stimulating emulation.

The report then referred to the needs of the profession in this matter, claiming that we have no literature on this subject worthy the name, or of the present state of knowledge. The lack of knowledge of pathology and etiology is palpably great, and largely due to the absence of text-books, and a want of appreciation of its importance. The idea that the teeth are parts of a vital organism, has no force in the minds of the profession. There has been no satisfactory solution of the daily problems presented in the mouths of patients; the accepted theories will fail to satisfy studious inquiry, and the conclusion is daily forced upon us that we have to deal with diseases governed by the same laws as those which preside over the whole body. The work needed should not only consider underlying principles, but their method of application. Very few in any profession have the ability to make proper application of general principles; those who can do so stand out in bold relief from the mass. The work should be comprehensive, clear, and concise, and apt in illustration. The work of Prof. Tyndall on Heat and Sound affords the best example of the required style; treating subjects hitherto imperfectly understood in such a manner that any ordinary mind can comprehend them.

The report then enlarged upon the fact that the etiology of dental caries, whether we accept the vital, the chemical, or the chemico-vital theory, is but imperfectly understood; no one nor all these will explain the causes. Predisposing causes are not a satisfactory explanation—there is something beyond. Teeth are daily witnessed that by this theory should be particularly subject to decay, but yet are retained in a comparatively sound condition, while those on the contrary quite the reverse, are sometimes hopelessly destroyed. These phenomena force upon us the conviction that dental caries is not simply a local disease, but a local expression of a constitutional disorder. Chemical action is the immediate cause, but this is only rendered possible by the constitutional derangement. If this be true, then constitutional disease must be the cause, and it should be amenable to constitutional treatment, and the time is coming when it will be demanded, as much as operative treatment is now. This leads us to affirm that the dental practitioner should be educated to the fullest extent in general medicine if he hopes to develop the highest beneficence, and this will at no distant day be demanded by the public.

The magnitude of the proposed work was then noticed; it must the result of immense labor, experiment and research, attended with peculiar difficulties. To such a work must be brought a mind well stored with knowledge of the principles and practice of medicine, and trained in observation and investigation, with almost infinite patience and diligence; and a power to cast aside all preconceived opinions. Your committee have apparently wandered, only to present some grounds for the position they assume in offering suggestions touching the literature of the future. The demand for such text-books, as are here referred to, they feel to be the great demand which now presses upon as, and which sooner or later must be satisfied. Your committee felt impelled by a sense of duty to say this much, and though their views are feebly and imperfectly presented, they hope their efforts may not prove entirely fruitless.

Dr. Francis thought every dentist who is competent to do so should write one or more articles a year for the dental journals. If articles were not accepted at first, the writers should not feel badly about it, but should try again.

Drs. Cook and Morrison were appointed a committee to conduct the newly elected officers to their seats.

Dr. Cushing, on taking the chair, thanked the association for the evidence of good feeling and confidence manifested in placing him in that position, and said that he would endeavor to preside with impartiality and satisfaction to the association.

Dr. Morgan, the retiring President, again thanked the association for the honor conferred upon him, and also for the courtesy extended to him during the past few days. He congratulated the body upon the harmony and good feeling that had prevailed, and upon the general character of the discussion. The papers read would do honor to any scientific body. The contrast between the present meeting and those a few years back deeply impresses him. He would also congratulate the body upon their newly elected officers. This association has exerted a powerful influence for good; local societies have sprung up; a more liberal spirit has been inaugurated. This society has a controlling influence on the literature of the profession in this and other countries The future is bright. The next twenty years will accomplish much more. He is glad to see the gray hairs present; it inspires him with high hopes for the future.

A resolution of thanks to the retiring and re-elected officers, for the successful discharge of their duties, was adopted.

Also, a resolution of thanks to the reporters present, and to the railroads for favors extended, was passed.

The Committee on Prize Essays had no report.

The minutes of the day's session were then read and approved.

A motion was carried instructing those holding any number of copies of Transactions to forward them at once to the Treasurer, as he frequently had calls which he was unable to supply.

A committee of ten was appointed to secure half fare rates to the next meeting, to consist of the officers of the Society.

Adjourned to meet first Tuesday in August, 1872, at Niagara Falls.—*Reported for "Cosmos," by C. Stoddard Smith, D.D.S.*

---

